# Niche differentiation and evolution of the wood decay machinery in the invasive fungus *Serpula lacrymans*

**DOI:** 10.1038/s41396-020-00799-5

**Published:** 2020-10-19

**Authors:** Jaqueline Hess, Sudhagar V. Balasundaram, Renee I. Bakkemo, Elodie Drula, Bernard Henrissat, Nils Högberg, Daniel Eastwood, Inger Skrede

**Affiliations:** 1 Department of Biosciences, University of Oslo, Oslo, Norway; 2 Department of Botany and Biodiversity Research, University of Vienna, Vienna, Austria; 3 Department of Soil Ecology, Helmholtz Centre for Environmental Research, UFZ, Halle (Saale), Germany; 4 Architecture et Fonction des Macromolécules Biologiques (AFMB), CNRS, Aix-Marseille University, Marseille, France; 5 INRA, USC1408 AFMB, Marseille, France; 6 Department of Biological Sciences, King Abdulaziz University, Jeddah, Saudi Arabia; 7 Department of Forest Mycology and Plant Pathology, Swedish University of Agricultural Sciences, Uppsala, Sweden; 8 Department of Biosciences, University of Swansea, Swansea, UK

**Keywords:** Molecular evolution, Fungal ecology, Comparative genomics

## Abstract

Ecological niche breadth and the mechanisms facilitating its evolution are fundamental to understanding adaptation to changing environments, persistence of generalist and specialist lineages and the formation of new species. Woody substrates are structurally complex resources utilized by organisms with specialized decay machinery. Wood-decaying fungi represent ideal model systems to study evolution of niche breadth, as they vary greatly in their host range and preferred decay stage of the substrate. In order to dissect the genetic basis for niche specialization in the invasive brown rot fungus *Serpula lacrymans*, we used phenotyping and integrative analysis of phylogenomic and transcriptomic data to compare this species to wild relatives in the Serpulaceae with a range of specialist to generalist decay strategies. Our results indicate specialist species have rewired regulatory networks active during wood decay towards decreased reliance on enzymatic machinery, and therefore nitrogen-intensive decay components. This shift was likely accompanied with adaptation to a narrow tree line habitat and switch to a pioneer decomposer strategy, both requiring rapid colonization of a nitrogen-limited substrate. Among substrate specialists with narrow niches, we also found evidence for pathways facilitating reversal to generalism, highlighting how evolution may move along different axes of niche space.

## Introduction

Adaptation to exploit a habitat can lead species to develop specialized phenotypes. Species vary greatly in the degree of specialization to environmental conditions, nutritional resources, and competitors they experience. Species specialized in inhabiting a narrow niche may face lower competition pressure [1], and they may be unable to adapt to changing environments due to their restricted range [2]. In turn, generalists with wide niches face more competition for resources and, in some cases, may experience trade-offs associated with being less well adapted to particular aspects of the environments they inhabit [3]. Niche breadth evolution therefore has important implications for evolutionary processes such as speciation and extinction, yet the underlying genetic architecture remains unresolved in many systems [4].

Fungi have adapted to colonize diverse habitats and their compact genome sizes make them ideal model systems to study the evolution of niche breadth in Eukaryotes on genome-wide scale. Many species are dependent on plants for their survival, either via biotrophic interactions (e.g., root and leaf pathogens, mycorrhizal fungi, and endophytes) or in the decay of dead plant material. Wood decomposition is dominated largely by Agaricomycete fungi [5] and constitutes an important part of the global carbon cycle. Exploitation of the plant cell wall (PCW) resource by these species provides a tractable system to study niche-driven specialism through genetic adaptation. PCWs of woody tissues are recalcitrant substrates made up of complex carbohydrates (cellulose, hemicellulose, and pectin) crosslinked to lignin, a polyphenolic substance that represents a strong barrier requiring a specialized decomposition machinery for microbes to access resources [6]. The evolution of lignin-depolymerizing class II peroxidases ~300 MYA probably led to the emergence of the white rot (WR) decay mode and facilitated the radiation of Agaricomycete fungi [7]. The brown rot (BR) nutritional mode, where decay-modified lignin is not catabolized, appears to have emerged independently from WR lineages multiple times, and is often associated with the decay of more lignin-rich softwoods [8]. Present day WR species generally have an expanded complement of peroxidases, associated oxidoreductases and carbohydrate active hydrolases to exploit the entire PCW resource [7, 9]. Whereas the BR mode is characterized by a refined suite of enzymes, where evidence strongly suggests the loss of class II peroxidase-driven delignification is replaced by a chelator-mediated Fenton (CMF) mechanism that modifies the lignin to access the carbohydrates of the PCW [10–12]. It is proposed that low pH iron oxidation by hydrogen peroxide generates reactive oxygen species that disrupt the lignin polymer releasing PCW carbohydrates for subsequent hydrolytic enzyme decomposition [13]. These processes are understood to take place in distinct stages, an early phase characterized by oxidative attack on the substrate, followed by a hydrolytic phase resulting in stage-specific expression of decay enzymes [14–16]. Similarly, nonenzymatic components of the decay machinery also show stage-specific expression, for example early decay in the BR fungus *Rhodonia placenta* includes a short window of high oxalate production [17] which may help to provide a chemically favorable environment for CMF reactions [18]. However, such detailed understanding of decay progression is currently limited to a handful of systems and classification of decay mode based solely on gene repertoires has proven challenging, highlighting a greater diversity of fungal decay strategies [19, 20].

Saprotrophic fungi offer tractable systems to allow the assessment of the additive effect of physiological and ecological factors in characterizing niche breadth and the mechanisms underlying species separation [21]. Fungal decay machineries show varying degrees of adaptation to different substrates, ranging from decay specialists restricted to single tree species, to generalists able to utilize both angiosperm and gymnosperm substrates [22]. Furthermore, the influence of ecological factors on the decay community structure is becoming apparent [23, 24]. Abiotic (water availability, temperature, pH) and antagonistic competition affect dispersal and structure of successional decay community members [25].

BR fungi in the Serpulaceae (Boletales) span a range of niche widths from substrate specialists with a narrow geographic range to a cosmopolitan generalist. *S. lacrymans* var. *lacrymans*, commonly known as dry rot, causes timber decay in houses. It is a niche specialist characterized by thick mycelial cords, relatively rapid primary decay habit with a strong substrate preference for spruce and low antagonistic ability [26]. It has a natural range in high-altitude regions in Asia from where it has spread to temperate and boreal regions world-wide [27]. Two separate invasions into the built environment have been suggested, one to Europe and one to Japan, where this species is largely restricted to houses and rarely found in nature [27, 28]. While European populations were likely established by a few founding members and have experienced a strong population bottleneck, the Japanese populations show much higher levels of genetic diversity [27, 28].

There are two variants of *S. lacrymans*: *S. lacrymans* var. *lacrymans* and *S. lacrymans* var. *shastensis* [29, 30]. The variant *shastensis* is found on large logs of *Abies magnifica* close to the treeline in the Cascade mountain range, but is not found in the built environment to our knowledge [31]. *S. lacrymans* var. *shastensis* provides an ideal comparator to var. *lacrymans* as it shares many of the niche characteristics, including a strong in vitro substrate preference for spruce and poor combative ability [26], yet these varieties split about 9 MYA ago [30]. *S. himantioides* is more commonly associated with natural environments and less frequently found in the built environment. This species is globally distributed and has a broad substrate range, associated with both hardwoods and softwoods [30, 31]. *S. himantioides* provides a more genetically diverse comparator and occupies a distinctive niche compared to its relatives, i.e., a generalist behavior with intermediate decay rates for a wider range of substrates [26, 32, 33]. It also is a stronger competitor suggesting that it may occupy later stages in the decay succession [26].

In this study, we used a combination of experiments, phylogenomic reconstruction, and expression profiling on three different substrates, *Picea abies* (spruce)*, Pinus sylvestris* (pine), and sucrose-based media (Czapek Dox) to establish the niche breadth of *S. lacrymans* variants *lacrymans* and *shastensis*, and *S. himantioides* and study the evolution of their decay machineries within this context. In order to survey variation within var. *lacrymans* from different invasive populations with contrasting levels of diversity we also de novo sequenced the genome of a Japanese strain to distinguish the European and Japanese var. *lacrymans*. Our analyses aimed to determine whether evidence supported a shift between generalist and specialist wood decay among the widely distributed *S. himantioides* that inhabits a broad host range, and the ancestor of vars. *lacrymans* and *shastensis* which are associated with narrow treeline ranges. Comparison of decay machinery with *S. himantioides* indicated an increased reliance on CMF and decreased reliance on PCW-degrading enzymes (PCWDEs) in *S. lacrymans* variants. However, decomposition ability and gene expression analysis on spruce and pine suggest a genetic basis for an underlying variability in decay between individuals of *var. lacrymans* and a possible route to reversal to generalism, but without the trade-off in decomposition rates seen in *S. himantioides*.

## Materials and methods

### Fungal material and experimental set-up

**Fig. 1:**
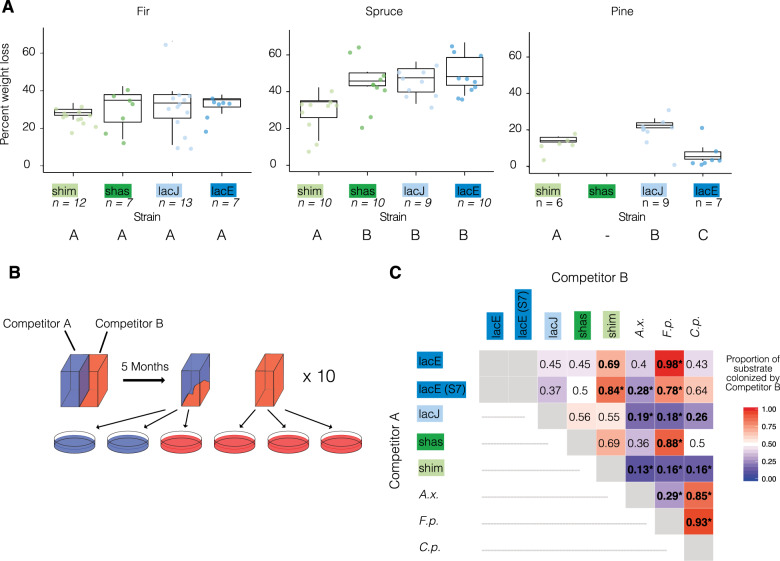
Strain phenotyping to determine niche breadths. **A** Weight loss of target strains on different wood types. Percent weight loss indicates the dry weight lost after 60 days of inoculation. Significant differences are indicated according to Kruskal–Wallis rank sum test (*P* < 0.05). **B** Experimental set up of the competition experiment. **C** Proportions of competitors recovered from substrate in a pairwise competition setup containing equal amounts of wood blocks inoculated with strain pairs. A proportion of 0.5 indicates no gain or loss of substrate, while proportions > 0.5 indicate substrate gain by competitor B and proportions < 0.5 indicate substrate gain by competitor A. Significant deviation from equal outcome (0.5) was tested using a *χ*^2^ goodness of fit test (df = 1). Bold: *P* ≤ 0.05; Bold*P < = 0.01. A.x. *Antrodia xantha*, F. p. *Fomitopsis pinicola*, C.p. *Coniophora puteana*. Sample sizes for each pair ranged from 19 to 48 observations (Supplementary Table S1).

Fungal strains SL200 (*S. lacrymans* var. *lacrymans*), SL198 (var. *lacrymans*), SHA17-1 (*S. lacrymans* var. *shastensis*), and MUCL38935 (*S. himantioides*) were maintained on Malt extract agar in the dark at 20 °C. Growth experiment data were taken from Balasundaram et al. [26] and supplemented with experiments for var. *lacrymans* strains SL198 and S7. Briefly, decomposition abilities for each strain were assessed by measuring percent weight loss of fir, spruce, and pine wood blocks 60 days after inoculation. Competitive capacities were determined by confrontation experiments on fir wood blocks. Pre-inoculated wood blocks were tied together and incubated for 5 months on average. For every pair, proportions of each strain re-isolated from ten replicate pairs of wood blocks were scored by subculturing on three plates per block, yielding up to 60 observations (2 wood blocks × 3 re-isolated plates × 10 replicates: Fig. 1B, Supplementary Table S1). Results were assessed for deviation from the initial proportion of 0.5 using *χ*^2^ tests. Both experimental procedures are described in detail in the Supplementary Material. To survey gene expression, we set up 150 mm diameter Petri dishes with 20 ml of Czapek Dox medium (SCD/control; [26]) or SCD without sucrose and glutamate but with a layer of wood shavings from either pine or spruce (pine—*P. sylvestris* and spruce—*P. abies*) in five replicates per treatment per strain. Wood shavings from untreated wood planks (H. C. Thaugland Trælastforretning, Oslo, Norway) were autoclaved twice at 121 °C for 30 min, leaving at least 24 h between treatments and stored at −20 °C until further use. The shavings were soaked in diH_2_O and autoclaved for a third time before ~2 g of wet weight wood shavings were spread evenly across the inoculated Petri dishes. Five fungal inocula of 5 × 5 mm were placed onto a 30 μm polyamide mesh (Sefar Nitex, Sefar, Heiden, Switzerland) and each plug was supplemented with 200 µl of 0.001% glucose solution. All treatments were grown at 20 °C in the dark for 30 days prior to harvesting.

### Nucleotide extraction, sequencing, and bioinformatic processing

Full details regarding DNA and RNA extractions, Illumina sequencing, de novo assembly and annotation of the SL198 genome and data processing for differential expression analysis are provided in the supplement (Supplementary Tables S2, S3 and Supplementary Data S1).

### Differential expression analysis

Differentially expressed genes were determined on a species-by-species basis using EdgeR v.3.16.5 [34] with the GLM approach and the quasi-likelihood *F*-test, based on an FDR-corrected *P* value of less than 0.05 and absolute log fold change greater than 1 (Supplementary Text, Supplementary Fig. S1). To determine the common set of genes induced on both types of wood (“core”) as well as those specific to spruce and pine, the following contrasts were implemented: (1) core = (spruce + pine)/2 vs. SCD, (2) spruce/pine = spruce vs. pine. Spruce-specific and pine-specific sets were determined by only retaining genes that were significantly upregulated in the respective wood type compared to the SCD control (contrasts 1a and 1b) as well as significantly different between the wood types in contrast 2 (Fig. 1B). Standardized expression levels for the purpose of visualization were obtained using the rlog function from DESeq2 [35] taking the median value within condition and obtaining a zero sum mean across the three conditions for each gene.

### Evolutionary analysis

Predicted proteomes of all four strains were clustered into gene families using OrthoMCL v.2.0.9 [36] with BLASTP *e*-value cutoff 1*e*^−5^ and percentMatchCutoff = 25. The inflation value was set to 3. For a total of 1010 non-trivial clusters (>3 sequences, >1 gene/strain) we used a full phylogenetic approach to determine orthology relationships and gene ages within the cluster (see Supplementary Material).

### Functional enrichment analysis

Enrichment of PFAM domains was conducted using Fisher exact tests corrected for multiple testing using the False Discovery Rate at threshold α = 0.05. GO term enrichment analysis was conducted using topGO v2.26.0 (89) with the “weighted01” algorithm and the Fisher exact test with *P* value cutoff 0.01.

## Results

### Growth experiments indicate niche differentiation

Decomposition experiments on different wood resources (pine, fir, and spruce) show distinct wood type - dependent responses among the four strains examined, with 0–69% of biomass (dry weight) consumed after 60 days growth and clear differences in decomposition among different species and wood types (Fig. 1A). The widely distributed generalist *S. himantioides* (strain MUCL38935 = shim) consumed all three wood types at moderate levels, on average reducing biomass by 15% on pine, 28% on fir and 34% on spruce. In contrast, the average weight loss on spruce for var. *lacrymans* strains (strain SL200 = lacE and strain SL198 = lacJ) and var. *shastensis* (strain SHA17-1 = shas) was significantly higher than for shim (Kruskal–Wallis rank sum test, *χ*^2^ = 15.471, df = 3, *P* value = 0.001455; Fig. 1A), with lacE causing up to 69% reduction. On pine, shas failed to establish under the conditions tested and lacE consumed on average less than 5% of the wood blocks. Strain lacJ caused significantly greater average weight loss on both spruce and pine (45% and 23% respectively; Fig. 1A). Testing two additional var. *lacrymans* isolates from Europe and New Zealand confirmed a strong preference for spruce, while indicating that weight loss on pine varied among individuals (Supplementary Text). No significant difference was found when species were grown on fir.

Antagonistic behavior of the *Serpula* strains profiled also supports niche differentiation between species. Each strain was competed against each other, a second *S. lacrymans* var. *lacrymans* European isolate (S7), and three common decomposer fungi (*Antrodia xantha, Fomitopsis pinicola*, and *Coniophora puteana*) using pre-colonized wood blocks of fir (Fig. 1B; [26]). The generalist decomposer shim emerged as a strong competitor as it defended its substrate against all other strains (Fig. 1C) and outcompeted five of the seven opponents (*χ*^2^ goodness of fit test, df = 1, *P* value ≤ 0.05). In contrast, poor competitors lacE and shas did not outcompete any strain tested. The second slac European isolate (S7) confirmed this trend, but was marginally more competitive, by outcompeting *A*. *xantha* (Fig. 1C). In contrast, lacJ showed strong antagonism, especially towards species outside the Serpulaceae. By the end of the experiment, lacJ significantly increased its proportion of substrate occupancy from 0.5 at the beginning of the confrontation to 0.81, 0.82, and 0.74 when confronted with *A*. *xantha*, *F*. *pinicola* and *C*. *puteana*, respectively.

### Transcriptomic profiling identifies conserved shift in decay machinery

**Fig. 2:**
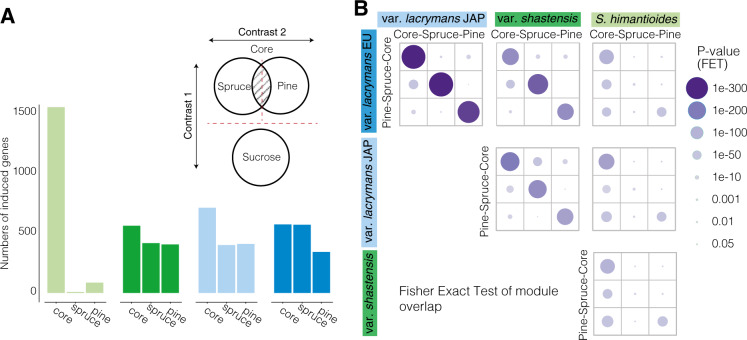
Wood type-dependent gene expression. **A** Design of the RNA-seq experiment to determine core wood, spruce- and pine-specific induced genes as well as the numbers of significantly upregulated genes for each module (FDR adj. *P* value < 0.01 and log fold change >1). **B** Significance of gene overlap between expression modules among different species. Size and shading of the circles correspond to the *P* values of the Fisher exact test (FET) for each module comparison. *P* values were corrected for multiple testing using the Benjamini–Hochberg procedure.

Specialization on spruce in vars. *lacrymans* and *shastensis* was mirrored by a conserved shift in transcriptional response when grown on spruce or pine wood shavings. We determined significantly upregulated transcripts (log2 fold change >1; FDR-adjusted *P* value < 0.01) on spruce and pine, as well as “core” wood-specific transcripts that were upregulated on both spruce and pine compared to sucrose (Fig. 2A). We refer to these sets of upregulated genes as spruce, pine, and core modules, respectively.

The generalist species shim expressed largely the same set of genes on both types of wood, with 1551 significantly upregulated core genes, 86 pine-specific genes, and only 8 spruce-specific genes (Fig. 2A, Supplementary Data S2). In contrast, lacE, lacJ, and shas showed distinguished expression responses to the different wood types with induction of 572 (lacE), 711 (lacJ), and 561 (shas) genes in the core module, 569 (lacE), 400 (lacJ), and 416 (shas) induced only on spruce, and between 342 (lacE), 409 (lacJ), and 405 (shas) genes specific to pine (Supplementary Data S3, S4, S5).

Genome-wide phylogenomic reconstruction inferred fine-grained homology relationships between genes in the four genomes (Supplementary Text, Supplementary Fig. S2). Comparison of one-to-one orthologs indicated that the partitioning of the transcriptomic response in the three *S. lacrymans* strains (lacE, lacJ and shas) is evolutionarily conserved and driven by overlapping gene sets in all three modules (Fig. 2B). Conservation between the three *S. lacrymans* strains and shim was strongest for the core module while there was a smaller, but significant overlap between the pine modules. Results also indicate significant overlap between the core module in shim and the spruce- and pine-specific modules in lacE, lacJ, and shas, supporting partitioning of a more general transcriptomic response to wood in shim into resource-specific transcriptomes in the three *S. lacrymans* strains.

### Functional signatures of resource-specific expression suggest distinct decay stages

**Fig. 3:**
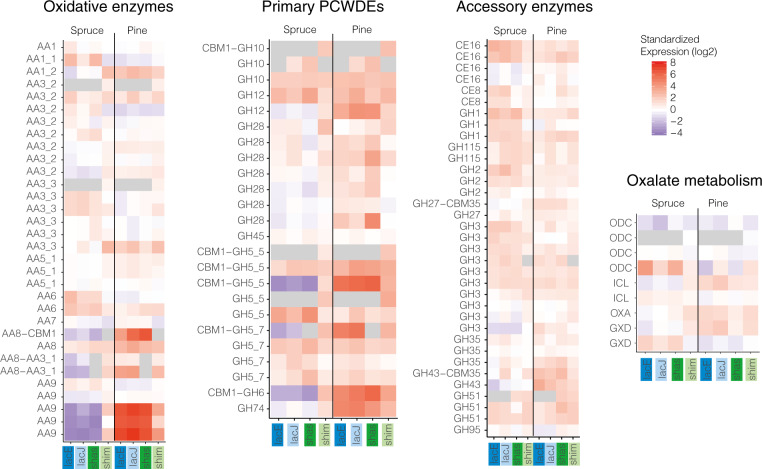
Standardized expression (mean centered log2 counts per million reads) of carbohydrate active enzymes involved in plant cell wall decomposition and oxalate metabolism. PCWDEs plant cell wall decomposition enzymes.

Carbohydrate active enzymes (CAZymes, including both hydrolytic and oxidative enzymes) known to be involved in the degradation of PCW material were mapped in each species into oxidative CAZymes, primary hydrolytic CAZymes acting on cellulose, hemicellulose and pectin, and accessory CAZymes metabolizing polysaccharides into simple sugars as well as assisting in PCW breakdown (Fig. 3, Supplementary Data S6). Many of the enzymes targeting crystalline cellulose (GH5_5 endoglucanases, GH6 cellobiohydrolase), hemicellulose (GH74 xyloglucanase, GH5_7 mannanase), and pectin (GH28 pectinase) were strongly upregulated on pine and repressed on spruce in lacE, lacJ, and shas. Similarly, several classes of oxidative enzymes known to be important for the depolymerization of lignocellulose, including three AA9 lytic polysaccharide monooxygenases, two AA8-AA3_1 cellobiose dehydrogenases with an iron reductase domain as well as an iron reductase domain fused to a cellulose-binding CBM1 module (AA8-CBM1) were strongly induced on pine and not detected on spruce in these strains. While some endoglucanases and mannases were induced on spruce, enzymes actively targeted to cellulose via a CBM1 were almost exclusively induced on pine in lacE, lacJ, and shas (Fig. 3, Supplementary Fig. S3). Although many of the key CAZymes mentioned above were also significantly upregulated on pine compared to spruce in shim (Supplementary Data S6), differences were subtle, and general patterns for both conditions closely mirrored the profiles found on pine in lacE, lacJ, and shas (Fig. 3, Supplementary Fig. S3).

Relatively few CAZymes were specifically induced on spruce for lacE, lacJ, and shas, between 12 and 18% of all wood-induced CAZymes, compared to a range of 47 to 53% on pine (Supplementary Data S6). Spruce-specific CAZymes were characterized as accessory CAZymes involved in digestion of oligosaccharides into simple sugars during more advanced decay stages, e.g., β-glucosidases (GH1 and GH3) and β-mannosidase (GH2), or debranching enzymes digesting hemicellulose side chains, e.g., CE16 acetylxylan esterase. No CAZymes were among the eight spruce-specific genes in shim.

Expression patterns of enzymes involved in oxalate metabolism also showed substrate-dependent differences (Fig. 3, Supplementary Data S6). We found strong upregulation of oxaloacetase (OXA) and one of two glyoxalate dehydrogenases, enzymes responsible for oxalate production, on pine in all four strains, while the same genes were repressed on spruce in lacE, lacJ, and shas. One of the four oxalate decarboxylases (ODC), which mediate the degradation of oxalate typical for later decay stages, was strongly repressed on pine in lacE and shas and induced on spruce in all strains (Fig. 3). In lacJ and shim, the same ODC was induced on pine which is consistent with more advanced decay of pine for both strains (Fig. 1A; [17]).

### Evolutionary analysis of wood-induced gene sets

**Fig. 4:**
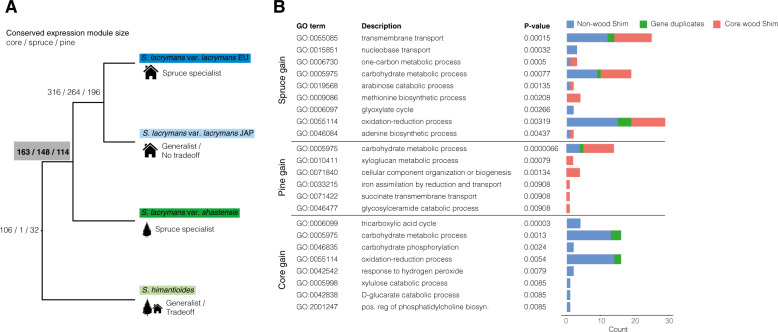
Evolutionary analysis of wood-induced gene sets. **A** Reconstruction of conserved expression modules at internal branches. Branch labels indicate the numbers of conserved genes in the core, spruce, and pine modules. **B** Functional enrichment of genes recruited to the spruce, pine, and core expression modules in the last common ancestor of vars. *lacrymans* and *shastensis* (highlighted in **A**). Bar charts on the right indicate the number of genes in each term and whether they are induced on wood in *S. himantioides* (red), not induced on wood in *S. himantioides* (blue) or genes duplicated in the LCA of vars. *lacrymans* and *shastensis* (green).

We mapped evolutionary changes to the wood decay machinery coinciding with the shift in decay capability between shim and the last common ancestor (LCA) of lacE, lacJ, and shas towards spruce (Fig. 4A). A total of 106 genes showed conserved induction on both wood types (“core”) in all four strains examined in this study, 32 showed conserved induction on pine only, while a single gene showed conserved spruce-specific induction in all strains. Spruce-specific genes increased disproportionally to 148 genes in the LCA of lacE, lacJ, and shas, while the core and pine modules gained 57 and 82 genes, respectively (Fig. 4A).

Functional enrichment analysis of the 147 genes added to the spruce module in the LCA of lacE, lacJ, and shas (Fig. 4B, Supplementary Data S7, S8) indicated broad changes to carbon and nitrogen metabolism. The term “carbohydrate metabolic process” GO:0005975 was significantly enriched, and genes driving enrichment of this term were predominately related to simple sugar metabolism and regulation of carbohydrate fluxes (Supplementary Data S8), mirroring targeted analysis of CAZymes (Fig. 3). Genes identified included enzymes involved in glycolysis, pentose-phosphate pathway, glyoxylate cycle, hemicellulose and pectin catabolism, and trehalose biosynthesis. A homolog of yeast SNF1 glucose-dependent catabolite regulation kinase was also identified.

Genes gained to the spruce module in the ancestor of lacE, lacJ, and shas were also enriched for uptake and metabolism of organic nitrogen. The strongest enrichment was for the term “transmembrane transport” GO:0055085 with 18 out of 25 associated genes involved in nitrogen transport e.g. oligopeptide transporters, permeases for nucleosides, purine and allantoate, three urea active transporters, and a nitrate transporter. Several terms involved in the biosynthesis of amino acids were also enriched (Supplementary Data S8). Many genes gained to the spruce module were not previously expressed on wood in shim (Fig. 4B, blue bars), and a large number of genes were recruited from the core module in shim to the spruce module in lacE, lacJ, and shas, suggesting a shift in the timing of expression of these genes (Fig. 4B, red bars, Supplementary Data S8). This concerned mainly genes involved in nitrogen metabolism and transport, xylose metabolism, and several GH18 chitinases.

Functional enrichment among genes gained to the pine module in the ancestor of lacE, lacJ, and shas included carbohydrate metabolism genes (GO:0005975), mostly CAZymes involved in the degradation of xyloglucan (GH12, GH31, GH27, GH16), pectin (GH28) and starch (GH13 and GH15), as well as an isocitrate lyase (EC 4.1.3.1), an enzyme linking the TCA and glyoxalate cycles. The term GO:0033215 “iron assimilation by reduction and transport” was also enriched, driven by an iron permease (Supplementary Data S7, S8). The majority of genes in the pine module of lacE, lacJ, and shas were recruited from the core module in shim, reflecting partitioning of the existing response to wood into early and late decay rather than recruitment of new gene sets (Fig. 4B, barplot). A notable exception to this were genes involved in pectin metabolism which were not induced on wood in shim (Supplementary Data S8).

Functional enrichment analysis of genes gained to the pine module in lacJ, a strong pine decayer, highlights genes putatively related to detoxification of plant extractives (Supplementary Data S9, S10). Enriched GO terms include “cellular amide catabolic process” GO:0043605, “oxidation-reduction process” GO:0055114, and “transmembrane transport” GO:0055085. The PFAM domains PF01476 and PF00067, encoding a LysM domain and Cytochrome P450s, respectively, were also significantly enriched.

### Loss of induction on wood in the LCA of vars. *lacrymans* and *shastensis*

**Fig. 5:**
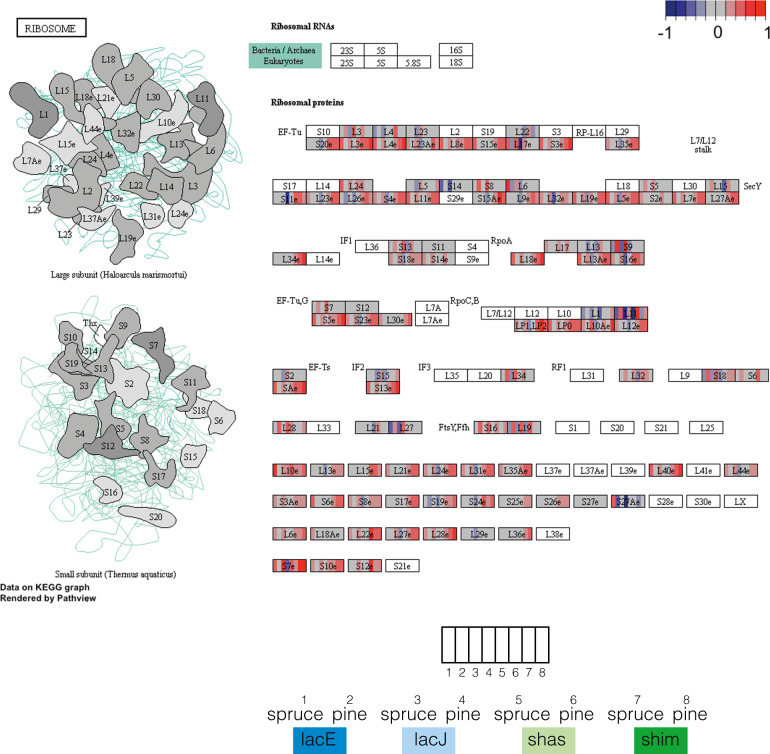
Expression levels of ribosomal proteins (KEGG 3010). Odd columns indicate spruce expression, even columns pine. Spruce/pine pairs are grouped by species as indicated in the legend. Plots were produced using PathView [58].

Transcripts that were induced on wood in shim, but where conserved induction on either type of wood was lost in the ancestor of lacE, lacJ, and shas, were strongly enriched for ribosomal proteins and those involved in ribosomal biogenesis (Fig. 5, Supplementary Fig. S4; Supplementary Data S11, S12). All of the 72 ribosomal proteins and snoRNAs identified (Supplementary Data S13) were significantly induced on both wood types in shim, while only 11 and 8 showed significant induction (core) in shas and lacE, respectively. Three and two showed core wood induction and spruce-specific induction, respectively, in lacJ.

The term “oxidation-reduction process” GO:0055114 was enriched among genes no longer induced on wood in lacE, lacJ, and shas, encompassing many cytochrome P450s and other putative components of the detoxification machinery. The term also includes one AA3_3 alcohol oxidase and four AA3_2 aryl alcohol oxidoreductases (Supplementary Data S12), all of which are H_2_O_2_ generating enzymes assisting oxidative degradation of PCW material [6]. Indeed, two of these genes were absent from the genomes of lacE, lacJ, and shas (see above, Fig. 3).

A streamlining of PCWDEs in lacE, lacJ, and shas was also suggested by targeted analysis (Fig. 3; Supplementary Data S6). Pine and core modules for shim included 118 CAZymes significantly induced during what we infer to be the active decay phase, when lignocellulose-targeted enzymes with CBM1s are expressed. In contrast, lacE, lacJ, and shas induced 98, 105, and 102 CAZymes, respectively, under the same conditions (Supplementary Data S6). Three CAZymes significantly upregulated in shim were lost from the genomes of lacE, lacJ, and shas (Fig. 3), including a GH10 endoxylanase with a CBM1 binding module (shim005171) and a GH5_5 endoglucanase (shim013482).

## Discussion

### Experimentally determined niche breadths mirror niche specialization in the wild

Decay experiments on different types of wood suggest that vars. *lacrymans* (lacE, lacJ) and var. *shastensis* (shas) are able to decompose at significantly higher rates compared to their close relative *S. himantioides* (shim), but that this is substrate-dependent (Fig. 1A, Supplementary Text). *S. lacrymans* var. *lacrymans* and var. *shastensis* inhabit a specialized niche in their natural ranges, where they grow on large logs at high altitude close to the tree line [27, 37]. Both varieties are pioneer species with a strong preference for *Abies* (fir) spp., although var. *lacrymans* has also been isolated from *Picea* (spruce) *smithiana* and less frequently from *Pinus* (pine) *wallichiana*, *Pinus sibirica* and *Cedrus deodara* [37]. *S. himantioides* which demonstrated a more generalist decay strategy (Fig. 1A; [26]), inhabits temperate forests globally and has been isolated from a variety of softwoods and hardwoods [33]. Competition experiments against other common forest fungi showed that the specialist decayers vars. *lacrymans* and *shastensis* are poor competitors, while *S. himantioides* showed an aggressive antagonistic behavior associated with widespread generalist species (Fig. 1B; [26]). Competition experiments using the Japanese var. *lacrymans* strain (lacJ), indicate that this strain also has superior combative abilities compared to lacE and shas (Fig. 1B). This suggests that the Japanese population may have an expanded niche compared to the European population (Fig. 1C). We hypothesize that this may be aided by greater genetic diversity in the Japanese population [27, 28].

### Wood type - specific responses highlight distinct decay stages

Expression profiles of PCWDEs indicate decay stage-specific responses between substrate types, in line with existing temporal models of BR decay [14–17] and experimentally measured decay abilities (Fig. 1A). Pine-specific induction of primary PCWDEs, especially those targeted directly to lignocellulose via CBM1s (Fig. 3, Supplementary Fig. S3), iron assimilation (Fig. 4B) and oxalate production (Fig. 3) indicate that all four strains are in early decay stages. While shim appears in a similar stage also on spruce, the downregulation of primary PCWDEs and induction of oligosaccharide processing enzymes (Fig. 3; Supplementary Data S8) indicate a more advanced decay on spruce for lacE, lacJ, and shas. These results highlight the need for increased emphasis on temporal aspects of wood decay in comparative studies. Synchronizing cultures on complex, heterogeneous substrates such as wood is challenging, but further development of space-for-time setups [14] and molecular markers for different decay stages will greatly facilitate this task.

### The decomposition machinery of each fungus reflects their respective niches

Evolutionary analyses of pine-induced genes and core modules, encompassing similar stages of decay and stage-independent genes, respectively, in all four strains, indicate a major shift in the transcriptomic response towards increased nitrogen use efficiency during wood decay in vars. *lacrymans* and *shastensis* (Figs. 3, 4). LacE, lacJ, and shas induced a smaller repertoire of CAZymes on pine compared to shim (Fig. 3, Supplementary Data S6), suggesting decreased reliance on nitrogen-intensive enzymatic decay. Three primary hydrolytic CAZymes, of which two (GH10-CBM1 and GH5-5) are among the top 10 induced genes in core and pine modules in shim, were lost from the genomes of lacE, lacJ, and shas altogether (Fig. 3, Supplementary Data S2). The strong induction of cellulose-targeted iron reductase during early decay in these strains instead supports a greater emphasis on CMF-mediated degradation of lignocellulose (AA8-CBM1, Fig. 3; [11]). The AA8-CBM1 gene is absent from *S. himantioides* (Fig. 3), but conserved among a wider set of fungi [26] and has been experimentally shown to participate in the depolymerization of lignin and cellulose [38]. The presence of AA8-CBM1 could provide greater CMF-mediated iron cycling capacity by adding to the previously described hydroquinone-driven mechanism caused by benzoquinone reducatase activity on elevated 2,5-dimethoxyhydroquinone levels associated with *S. lacrymans* degraded wood [39]. Reciprocal gene losses of key decomposition genes in the *S. himantioides* lineage as well as the LCA of *S. lacrymans* varieties underline that based on the present data it is unclear whether substrate generalism is ancestral to the Serpulaceae or a derived characteristic of *S. himantioides*. This subject will require further investigation.

*S. lacrymans* produces copious amounts of oxalic acid compared to both distantly-related BR species and close relatives *S. incrassata* and *C. puteana* [40, 41]. Oxalic acid solubilizes iron from Fe(oxyhydr)oxide and chelates it at low pH, as is found in close proximity to the fungal hyphae [42, 43]. Consistent with a role for oxalate in iron accumulation and transport, high oxalate concentrations in *S. lacrymans* were linked to the ability to accumulate large amounts of iron in wood substrates [40, 41] which in turn may facilitate the above discussed CMF-based degradation [18]. Wood is an extremely nitrogen-poor substrate with a C:N ratio of up to 1250:1 [44], e.g., compared to most European forest soils which have C:N ratios of between 16:1 and 44:1 [45]. Transcription and translation of genes encoding the molecular machinery required to exploit woody substrates use relatively large amounts of cellular nitrogen [46]. The shift in decay mechanisms from a more nitrogen-intensive enzymatic strategy (as in shim) to one that leverages products of high carbon flux, such as oxalic acid (lacE, lacJ, and shas), therefore may present an adaptation to nitrogen-limited substrates.

Optimization of nitrogen use in lacE, lacJ, and shas is also reflected by increased partitioning of carbon and nitrogen uptake and metabolism in space and time (Figs. 3, 4B, 5). We found a subtle but coordinated reduction in expression of ribosomal proteins during enzymatic decay in lacE, lacJ, and shas, suggesting reduced overall protein production during wood decay (Fig. 5, Supplementary Data S13). Even small changes, such as alteration in codon usage in highly expressed genes, can have a large impact on the nitrogen budget of an organism [47], highlighting the potential significance of these changes to nitrogen use efficiency. Similarly, we found recruitment of genes involved in nitrogen transport and metabolism from the core module in shim to the spruce module in the ancestor of lacE, lacJ, and shas (Fig. 4B), suggesting a shift to a later decay stage and/or greater fine-tuning to the composition of the substrate compared to shim.

Resource-optimized decay and nitrogen transport may also enable vars. *lacrymans* and *shastensis* to rapidly colonize and thereby monopolize its substrate which can serve as a defense strategy for a pioneer species with limited combative ability [48]. To this end, upregulation of pectin metabolism and degradation of pit membranes during early decay in lacE, lacJ, and shas (Supplementary Data S8) also facilitates rapid advancement of colonizing hyphae between plant cells, in particular in conjunction with increased oxalic acid production [49]. Pectin in pit membranes is complexed with calcium ions, and oxalic acid has been shown to chelate Ca^2+^ from pectin, rendering it more amenable for hydrolytic degradation [49]. This is consistent with the formation of calcium:oxalate crystals around pit membranes during decay by *S. lacrymans* [50].

### Decay machineries underpin substrate generalism

The shift to increased reliance on CMF in the LCA of vars. *lacrymans* and *shastensis* appears to represent a specialization for *Picea* spp. and *Abies* spp. as well as a small number of species from the genus *Pinus* (pine; see above). These species all share the common feature of low heartwood extractive content, in particular with respect to resin acids and pinosylvin stilbenes [51]. Differing extractive content may explain the substrate-dependent variation in decay rates and the poor decomposition rates of Scots pine wood by lacE and shas, in particular (Fig. 1). Pine wood is generally found more recalcitrant to decay than spruce in part due to extractives [52, 53]. Pinosylvins, the primary extractives found in Scots pine, are strong antioxidants and can inhibit BR decay by scavenging free radicals, preventing CMF reactions [54]. However, the enzymatic components of the BR decay machinery appear to be less affected by pinosylvins [55] and we hypothesize that the increased reliance on enzymatic decay in shim allows for more rapid decay of pine compared to the more CMF-heavy strategy of lacE and shas (Figs. 1A, 3).

In contrast, the increased weight loss caused by lacJ on pine compared to lacE, shas and shim may be due to a superior ability to detoxify pinosylvins or other inhibitory defense chemicals by this strain (Fig. 1A). Indeed, we found many cytochrome P450s and oxidoreductases among the pine-induced genes in lacJ but not lacE (Supplementary Data S9, S10). Cytochrome P450s have been found to degrade stilbenes in the BR *R. placenta* [56, 57] and are frequently implicated in response and adaptation to different wood types. Expansion of the detoxification enzymes, both by gene duplication and recruitment of existing genes, therefore provides an alternative pathway to substrate generalism, and apparently without the trade-off associated with having a reduced resource-adapted decay machinery (Fig. 1A, C).

Taken together, our results provide a framework for understanding the evolution of fungal decay machineries in response to substrate and habitat pressures. Our conclusions are limited by the small number of isolates used in this study, and a more fine-grained mapping of niche breadths will require additional experiments, using a broader range of strains, substrates, and competitors, chosen to reflect the different habitats that these species are found in. Nevertheless, we discovered an evolutionarily conserved shift in decay strategy that coincides with increased mass loss and a more specialized niche. Integrative comparative systems, combining growth experiments and evolutionary genomic approaches provide powerful tools to understand how eco-evolutionary feedback mechanisms shape genome evolution and increasingly complex systems. Nitrogen limitation, substrate range, and fungal defense strategy in particular emerge as likely drivers shaping the decay machineries of *Serpula* spp., highlighting the importance of considering multiple axes in a dynamic niche space when interpreting genomic data.

## Supplementary information

Supplementary Material

Datasets S1-S13

## Data Availability

Raw reads and assembly of the SL198 strain, as well as RNA-seq data were deposited at NCBI under Bioproject ID PRJNA655420. Updated genome annotations, functional annotations, alignments, and gene trees underlying the phylogenomic reconstruction were deposited on Dryad 10.5061/dryad.4f4qrfj93. Supplementary Information is available for download on the ISME website.
