# Supplementary material for: Niche differentiation and evolution of the wood decay machinery in the invasive fungus Serpula lacrymans
Source: ISME J. 2020 Oct 19;15(2):592–604. doi: 10.1038/s41396-020-00799-5 (PMC8027034; doi:10.1038/s41396-020-00799-5)
Supplement: Supplementary file 1 — Supplementary Material [file 41396_2020_799_MOESM1_ESM.pdf]

Supplementary Material

Niche differentiation and evolution of the wood decay machinery in the invasive fungus *Serpula lacrymans*

Jaqueline Hess[1,2,8], Sudhagar V Balasundaram[1], Renee I Bakkemo[1], Elodie Drula[3,4], Bernard Henrissat[3,4,5], Nils Högberg[6], Daniel Eastwood[7], Inger Skrede[1]

[1]Department of Biosciences, University of Oslo, Norway, [2]Department of Botany and Biodiversity Research, University of Vienna, Austria, [3]Architecture et Fonction des Macromolécules Biologiques (AFMB), CNRS, Aix-Marseille University, Marseille, France, [4]INRA, USC1408 AFMB, Marseille, France, [5]Department of Biological Sciences, King Abdulaziz University, Jeddah, Saudi Arabia. [6]Department of Forest Mycology and Plant Pathology, Swedish University of Agricultural Sciences, Sweden, [7]Department of Biosciences, University of Swansea, UK, [8]Department of Soil Ecology, Helmholtz Centre for Environmental Research, UFZ, Halle (Saale), Germany.

Supplementary Text

1) Growth experiments

Decomposition abilities were determined by inoculation on *Pinus sylvestris* (pine), *Picea abies* (spruce) and *Abies lasiocarpa* (fir) wood blocks as described in Balasundaram et at. [1]. Briefly, 2 x 2 x 1 cm wood blocks were dried at 40 °C for three days, numbered and weighed. Wood blocks were autoclaved three times at 24 h intervals and stored frozen until further use. Prior to inoculation, wood blocks were soaked in sterile diH_2O for 24 h. Ten replicates per strain and wood type were set up by placing 0.5 x 0.5 cm mycelial plugs directly onto the wood blocks. Wood blocks were placed into sterile 100 mL plastic containers filled with 20 g of sterile perlite and left to grow for 60 days at 20 °C in the dark. At harvest, mycelium was removed from the outside of the wood blocks and blocks were dried at 40 °C for three days prior to weighing. Statistical significance of the differences of weight loss of the decayed wood blocks among strains on each substrate were assessed using Kruskal-Wallis rank sum tests.

Competitive behavior was assessed by confrontation experiments on fir wood blocks, also following Balasundaram et at. [1]. Wood blocks were prepared for inoculation as described above. For each strain, a sterile 10 x 9 x 7 cm plastic container was filled with 40 mL of 2%

Malt Extract Agar (MEA) and inoculated with seven 0.5 x 0.5 cm mycelial plugs. Thirty-five wood blocks were added to each box and incubated at 20 °C in the dark for two months until all wood blocks were well inoculated. Strains were paired by scraping off the mycelium on the surface of respective wood blocks and placing the 2 x 2 cm sides of two wood blocks together with cut tracheid ends facing each other, and fixing them together using a sterile rubber band. A total of ten replicates for each combination of strains was set up. After approximately five months of growth the dominant strain in each wood block was determined by sampling from three central sampling points along each wood block that had had surface mycelia removed and split. Therefore, six cultures were prepared on MEA for each paired interaction. Strain identity was determined either by visual assessment or by producing a sanger sequence of the ITS region as a barcode. Please refer to Balasundaram et al. [1] for additional details. Results were scored as the proportion of samples from which each strain was recovered in a particular confrontation and statistical significance of deviation from the initial proportion of 0.5 at the start of the experiment was assessed using Person χ^2 goodness of fit tests.

2) RNA extraction, library preparation and sequencing

Samples were frozen under liquid nitrogen and ground to a fine powder using a pestle and mortar. CTAB buffer (2% CTAB, 2% PVP, 100 mM Tris-HCL, 2 M NaCL, 25 mM EDTA, 300 µL/15 mL beta-mercaptoethanol) was added and incubated at 65 °C for 15 minutes, before two extractions with one volume of chloroform for 10 mins. RNA precipitated overnight in 1/3 volume 8 M LiCl was centrifuged at 10,000 RPM for 40 mins at 4 °C and resuspended in molecular grade water. Additional RNeasy Mini Kit and RNase-Free DNase Set (Qiagen, Hilden, Germany) clean-up steps were included.

RNA concentrations were adjusted to 10 ng/µl following Qubit (Thermo Fisher, Waltham, MA, USA) quantification and sequencing libraries were prepared using the TruSeq RNA Library Prep Kit v2 (Illumina, San Diego, CA, USA) following the manufacturer's protocol. Finished libraries were pooled and sequenced on an Illumina NextSeq 500 instrument at the Norwegian Sequencing Centre. For each species, one of the four SCD replicates was sequenced in 150 bp PE mode to aid improvement of genome annotations while the remaining libraries were sequenced in 75 bp single-end mode.

2) Genome sequencing of *S. lacrymans* var. *lacrymans* SL198 (lacJ)

To facilitate full phylogenomic analysis, we sequenced and assembled the genome of the Japanese var. *lacrymans* strain SL198. DNA of the strain SL198 was extracted and sequenced according to Balasundaram et al. [1]. The preparation of an Illumina 108 bp paired-end (PE) library and sequencing of one lane of Illumina GAII was performed at SNP&SEQ Technology Platform in Uppsala, Sweden, yielding a total of 24,893,665 paired-end reads. Raw reads were trimmed using Trimmomatic tools v.0.35 [2], and the genome was assembled with Velvet de novo assembler [3], using the same settings as for SL200 and SHA17-1 [1]. This resulted in an assembly comprising 1,827 scaffolds > 1000 bp and an N50 of 54kb. The full assembly statistics are shown in SI Table S2. Genome completeness and redundancy were assessed using BUSCO v.2 [4] with the Basidiomycota reference database. BUSCO results for the SL198 assembly were comparable with the previously assembled genomes, suggesting a nearly complete gene space (97% of full length BUSCO genes recovered) with low levels of redundancy (0.4% of duplicate BUSCO genes).

3) Genome annotation improvement

We used the "Just Annotate My Genome" pipeline https://github.com/genomecuration/JAMg to produce updated annotations for the genomes sequenced in (1) and the newly sequenced SL198. The full procedure is described here: https://github.com/JackyHess/Fungal_genome_annotation. Briefly, RNA-seq libraries were aligned to the genome using Hisat2 v2.0.4 [5] with the options --dta --min-intronlen 20 --max-intronlen 300. Trinity v.2.2.0 [6] was used to calculate both *de novo* and reference-based transcriptome assemblies, with the option --jaccard_clip to reduce the number of fused transcripts. Transcriptome assemblies were fed to PASA [7] to generate a comprehensive transcript database. Gene models were predicted using Augustus v.3.0.2 [8] and GeneMark-ET v.4.32, both packaged into the BRAKER1 software [9], and CodingQuarry v.1.2 [10]. Finally, EvidenceModeler [7] was used to combine gene predictions into a set of proposed high quality gene models for each locus. In order to ensure maximum coverage of the gene space, we screened individual *de novo* predictions, as well as the initial set of gene predictions from [1] for full length gene models not overlapping the high quality set. Putative non-overlapping gene models were kept if their predicted coding sequence was longer than 300 bp and they

were expressed with at least 2 fragments per kilobase per million reads (FPKM) in either of the three conditions.

Genome annotations yielded between 12,800 and 16,174 gene models (SI Table S3). Of these, between 68.8% and 80.8% were expressed with an FPKM of at least 1 among the sequenced samples. A BUSCO run in 'protein mode' produced completeness values between 89.7% and 97.4%, mirroring estimates calculated based on the unannotated genome sequences (SI Table S3). This suggests that annotations are representative of the assembled gene space.

Predicted proteins were annotated for functional domains using InterProScan v5.4-47.0 [11] with the options: -iprlookup -goterms -pa -dp. PFAM annotation was run using hmmscan from the HMMER package v3.1b2 [12] with e-value cutoff 0.1 and PFAM version 27.0 [13]. Finally, proteins were assigned to conserved orthologous groups using eggNOGmapper [14] with eggNOG database version 4.5.1 [15] in one2one ortholog mode and the following search settings:-d fuNOG. For GO term enrichment analysis non-redundant annotations from InterProScan and eggNOG were merged. CAZyme detection and assignment to families were done using the standard procedure used for the daily updates of the CAZy database (www.cazy.org, [16]).

Using the procedure outlined above, we were able to assign approximately 70% of genes to eggNOG ortholog groups across all strains, while 47-48% of genes were associated with one or more GO terms (SI Table S3).

4) RNA-seq experiment data processing and QC

Raw reads were processed with Trimmomatic v0.35 [2] to remove adapter sequences and poor quality bases, using the following settings: ILLUMINACLIP:TruSeq3-SE:2:30:10 MAXINFO:30:0.4 MINLEN:30. Single-end libraries were aligned to each reference genome using 2-pass mapping with STAR v.2.5.2a [17], setting alignIntronMax to 750. QoRTs v.1.1.8 [18] was used for QC and to generate counts per gene for downstream analysis.

Sequencing of our RNA-seq libraries resulted in between 35 million and 69 million raw reads per library, with alignment rates between 88% and 98% with the exception of one shim outlier with 56% alignment rate (SI Data S13). Gene space covered in the individual genomes ranged

from approximately 70% in shim to 84% in lacE and was stable among conditions and replicates within each strain irrespective of sequencing depth, suggesting that we achieved good coverage of the active transcriptomes in the conditions assayed. Principle component (PC) analysis of count data using DESeq2 [19] revealed strong clustering according to substrate (SI Fig. S1). PC1 separates SCD and sawdust samples and PC2 separates the different wood types for all four strains. Total percent variation explained by the first two PCs ranged between 86% (lacJ) and 99% (shim), suggesting that most variation is driven by our experimental design.

5) Phylogenomic reconstruction

In order to reconstruct evolutionary histories of gene families and identify orthologous genes in the four genomes studied, we implemented a phylogenomic reconstruction pipeline. Clustering of amino acid sequences into gene families resulted in a total of 10,584 clusters (see Dryad Annotation File). Of these, 7,316 were shared among all strains (SI. Fig. S2A). The percentage of protein coding genes clustered into gene families was 81% (10,418), 78% (10,442), 79% (10,171) and 76% (12,268) for lacE, lacJ, shas and shim, respectively, indicating similar proportions of singletons under our strict clustering settings.

For each cluster, protein sequences were aligned using PRANK v150803 [20] with default parameters. Poor quality regions were removed from alignments using TCS scores as implemented in TCoffee [21], filtering residues with scores < 4. The best-fit model of evolution was determined for each trimmed alignment using ProtTest v3.4.2 [22] and the following settings: -JTT -WAG -G -S 1 –AIC. Maximum Likelihood (ML) trees were estimated with RAxML v8.2.10 [23] using the model of best fit and the options –f a –N 10. Gene trees were improved using the species-tree aware error correction tool TreeFix [24] with the "long" settings --nquickiter=100 --niter=1000 and the best model of evolution for each alignment. Finally, corrected gene trees were reconciled with the species tree using DLCpar [25] setting --max_dups=-1 and --max_losses=-1. Orthology relationships and gene copy ages were parsed from reconciled gene trees with a Python script leveraging tools from the ETE3 library for phylogenetic analysis [26]. The script is available here: https://github.com/JackyHess/Serpula_transcriptomics.

Inference of gene trees and reconciliation with the species tree (SI Fig. 2B) yielded results similar to the birth-death model-based analyses in Balasundaram et al. [1]. Gene content evolution in both *Serpula lacrymans* varieties is characterized by gene space reduction with the number of losses outweighing the numbers of duplications. A notable difference to the results presented in Balasundaram et al. [1] is the small number of gene losses inferred in the ancestor of vars. *lacrymans* (lacE and lacJ) and *shastensis* (shas; SI Fig. 2B). Using the phylogenetic approach, we only inferred 10 losses on this branch and approximately ten times the number of duplications, while the model-based approach resulted in 332 losses and only five duplications [1]. This discrepancy is likely due to an underestimation of the number of losses at this node using the phylogenetic approach since singleton genes in *Serpula himantioides* (shim) would have been excluded from sequence analysis. Alternatively, it is possible that the model-based approach underestimated duplications on the shim lineage and therefore overestimated losses in the ancestor of lacE and shas. In order to resolve this pattern, full phylogenomic studies with a wider taxonomic distribution are required.

Since a major shift in decay ability and transcriptomic response to wood occurred in the ancestor of lacE, lacJ and shas we investigated genes duplicated on this branch. Functional enrichment analyses suggest an enrichment for oxidative enzymes, in particular Cytochrome P450s (GO:0055114 'oxidation reduction process'), H_2O_2 detoxifying enzymes (GO:0042744 'hydrogen peroxide catabolic process') and a family of beta-glucosidases in CAZy family GH1 (GO:0030245 'cellulose catabolic process').

6) Variation between strains

Profiling of two additional var. *lacrymans* isolates confirmed significantly higher mass loss on spruce for all profiled *S. lacrymans* strains, both compared to shim as well as other common BR fungi ([1]; Skrede et al., *in review*). While mass loss on pine was consistent within isolates, strains differed in their ability to decompose pine, indicating that var. *lacrymans* individuals across populations include both spruce specialists and those with more generalist decay abilities but a clear spruce preference. Our choice of strains here therefore represents two decay profiles segregating within var. *lacrymans* rather than a divergence between populations.

Supplementary Tables:

Table S1: Sample sizes competition experiment
Table S2: Assembly stats
Table S3: Annotation stats

Supplementary Data: [provided as Excel file]

Data S1: RNA-seq datasets
Data S2: shim induced genes
Data S3: lacE induced genes
Data S4: lacJ induced genes
Data S5: shas induced genes
Data S6: CAZyme data
Data S7: LCA(lac_shas) gain modules
Data S8: LCA(lac_shas) gain functional enrichment
Data S9: lacJ gain pine
Data S10: lacJ gain pine enrichments
Data S11: LCA(lac_shas) loss
Data S12: LCA(lac_shas) loss functional enrichment
Data S13: Ribosomal genes

Supplementary Figures

Figure S1: RNAseq counts – PCA
Figure S2: Clustering statistics and phylogenomic analysis
Figure S3: Absolute expression, core PCWDEs
Figure S4: Ribosomal biogenesis pathway

Supplementary Figures:

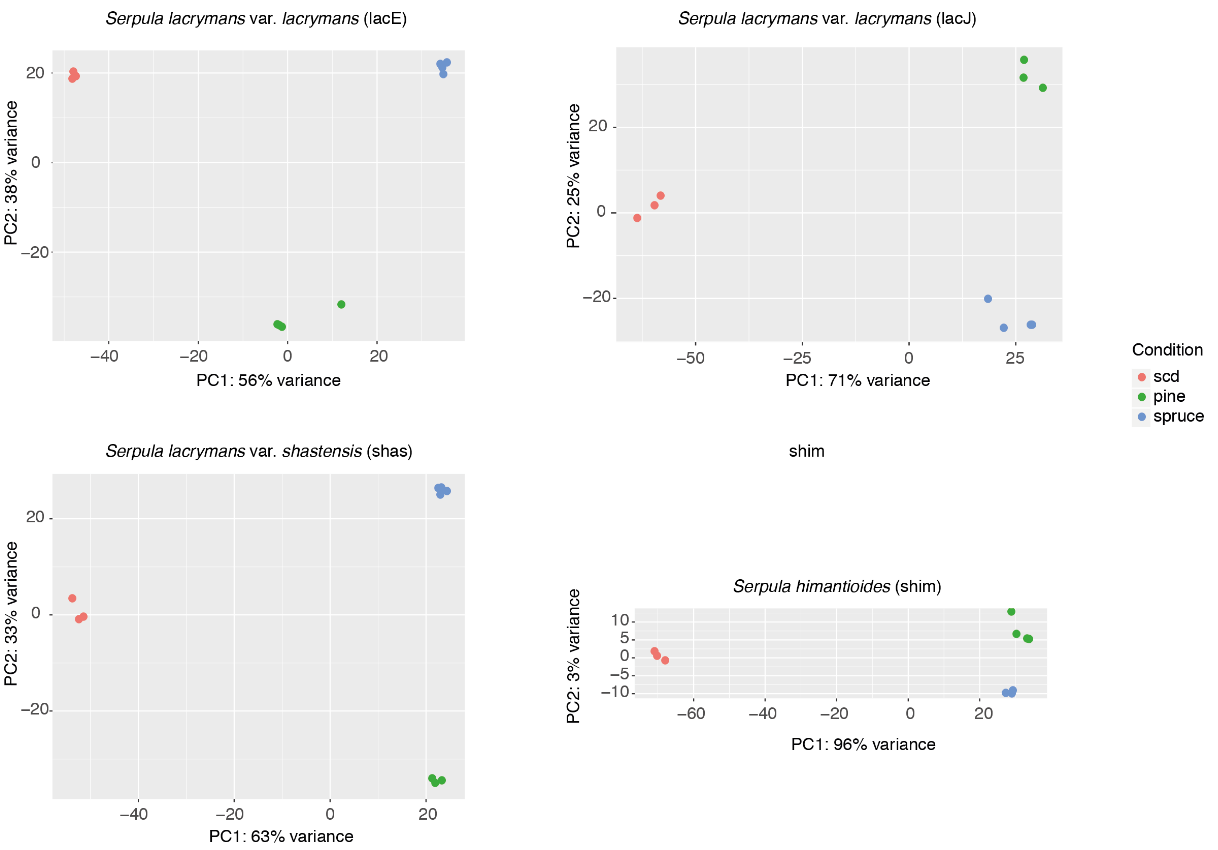

Figure S1: Principle component analysis of RNAseq count data according to condition.

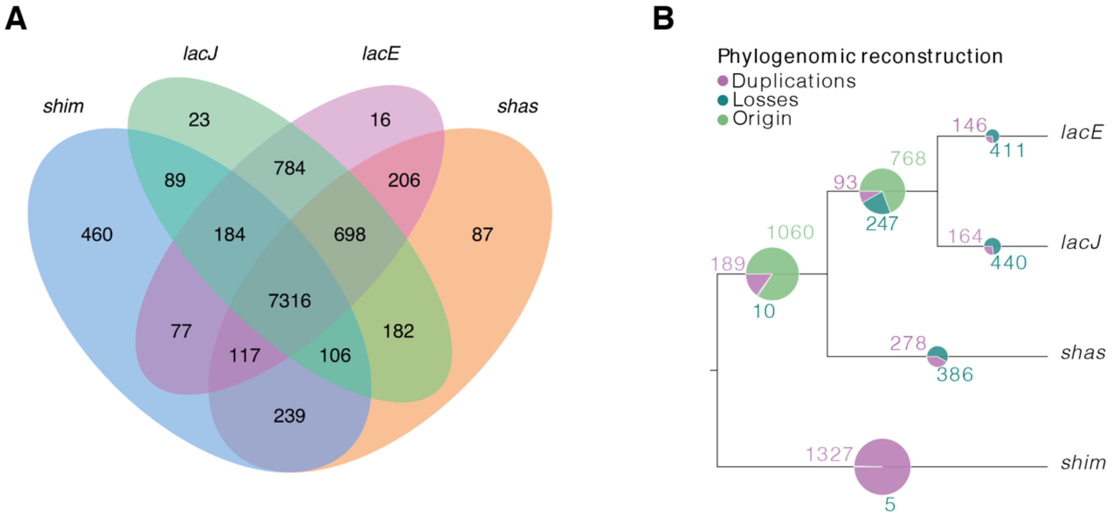

Figure S2: Phylogenomic analysis. (A) Clustering of genes into ortholog groups. (B) Numbers of duplications (purple), losses (dark green) and gene families originating at each respective node (light green) as inferred from reconciliation of gene trees with the species tree. Gene family origins indicate gene families only present in the subtree below the node. They can be the result of a variety of processes: i) gene loss other parts of the tree, ii) rapid divergence of homologs in different parts of the tree (with or without gene duplication on the branch in question), iii) horizontal gene transfer, iv) de novo gene creation or v) annotation error.

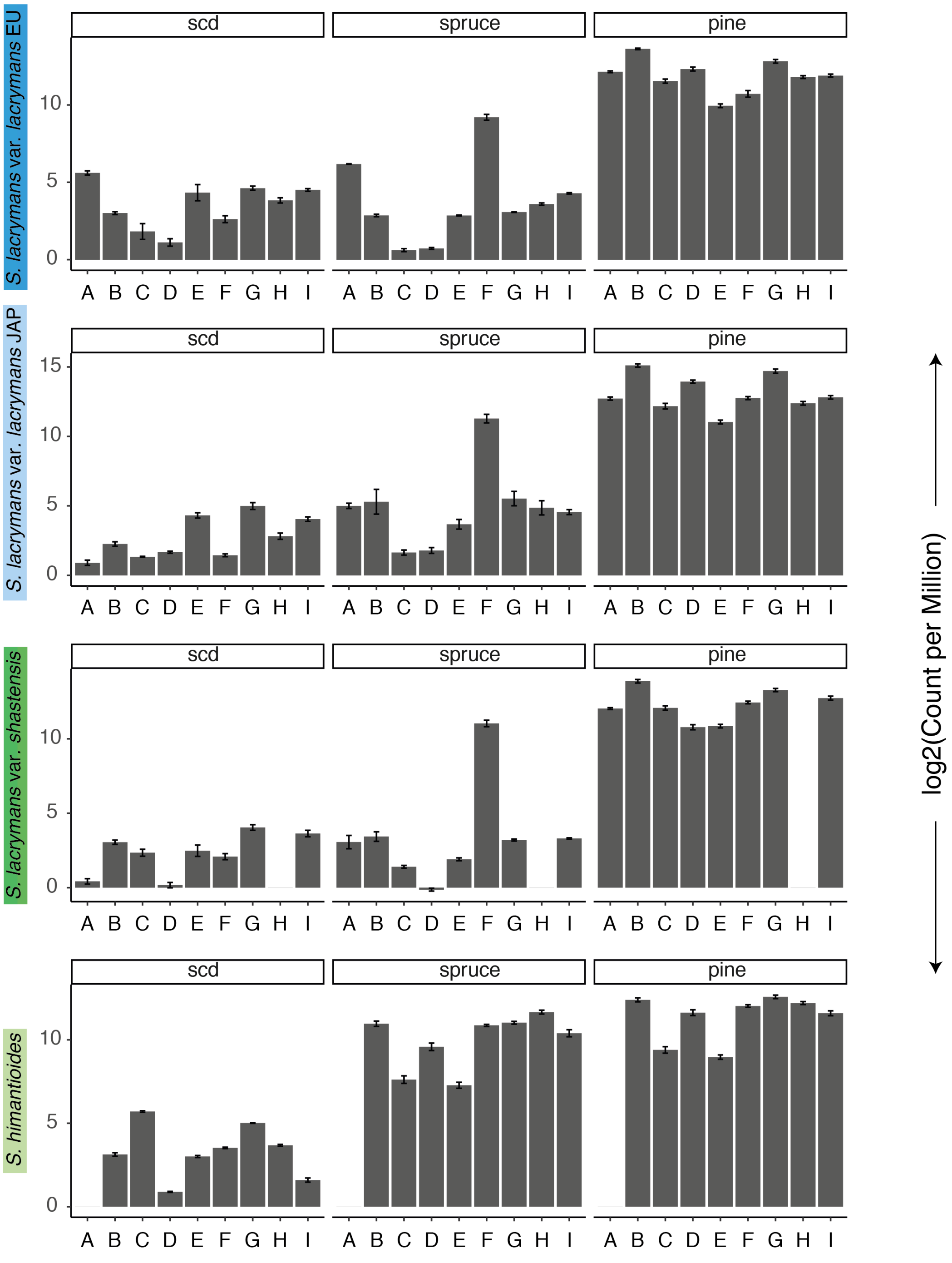

Figure S3: Expression levels of strongly regulated PCW-degradation CAZymes in count per million (log2 scale). A: OG2859_1; AA8-CBM1. B: OG2064_1; AA9. C: OG5953_1; AA9. D: OG6649_1; AA9. E: OG2574_1; GH131-CBM1. F: OG233_1; CBM1-GH5_5. G: OG233_2; CBM1-GH5_5. H: OG7948_1; CBM1-GH5_7. I: OG2303_1; CBM1-GH6.

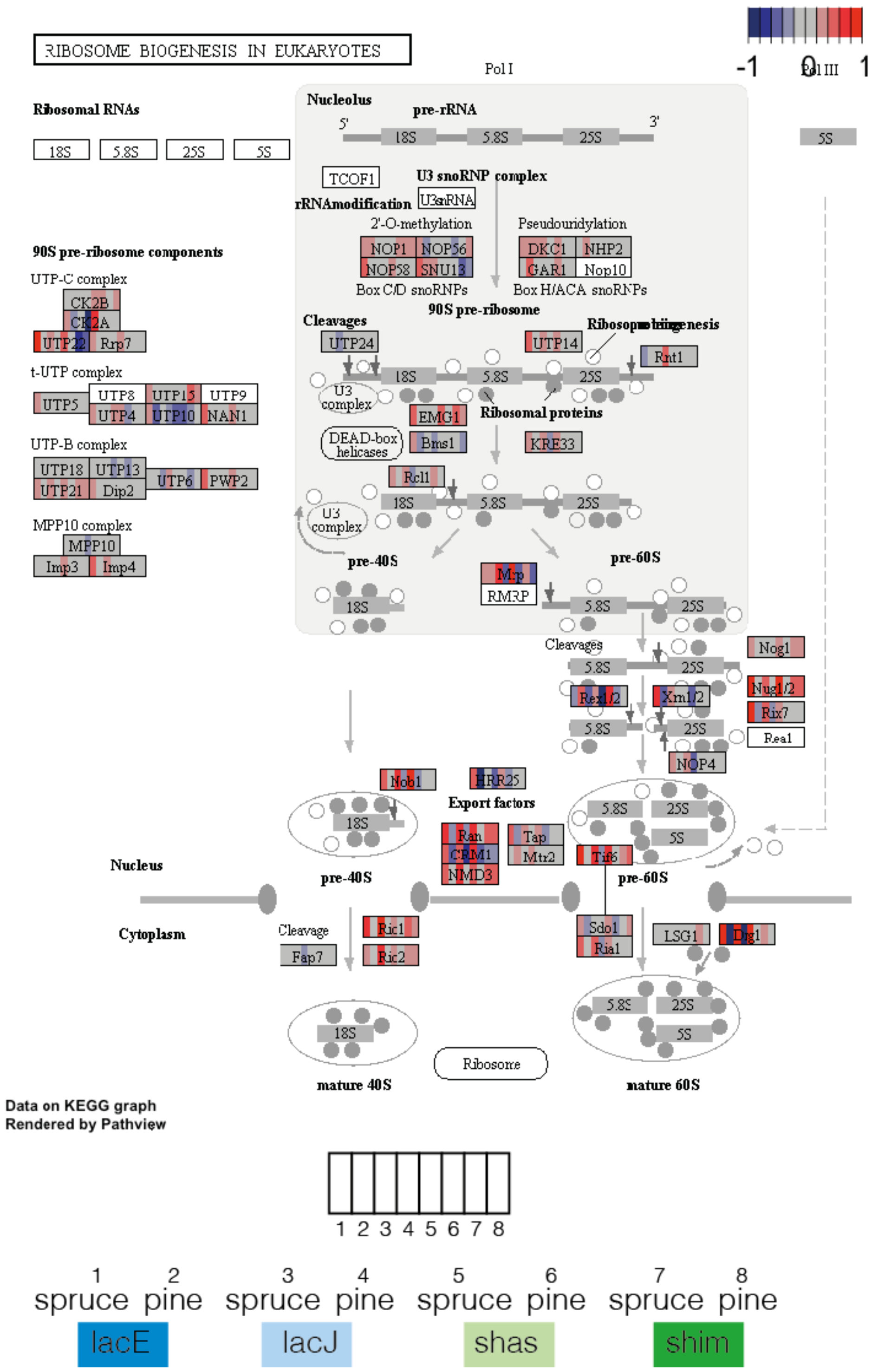

Figure S4: Ribosomal biogenesis pathway in Eukaryotes (KEGG 3008) with standardized expression values on spruce and pine according to the above key. Figure plotted using PathView (11).

Supplementary Tables:

Table S1: Sample sizes competition experiment

Strain	lacE	lacE (S7)	lacJ	shas	shim	C.p.	A.x.
lacE							
lacE (S7)							
lacJ	28	19					
shas	20	24	32				
shim	45	25	43	27			
C.p.	43	38	25	34	45		
A.x.	45	34	37	38	44	39	
F.p.	46	46	37	45	48	29	48

Table S2: Assembly statistics

Strain	Assembly size	# Scaffolds	N50	BUSCO complete %	BUSCO duplicated %	Reference
SL198	39,437,580	1,827	53,739	97	0.4	This study
SL200	37,393,313	1,529	59,716	97.4	0.4	Balasundaram et al. 2018
SHA17-1	38,078,689	1,170	92,207	96.9	0.4	Balasundaram et al. 2018
MUCL38935	45,914,007	4,826	19,719	88.6	0.6	Balasundaram et al. 2018

Table S3: Annotation statistics

Strain	# Gene Models	Expressed Gene Models (%)*	BUSCO complete %	GO annotation (%)	EggNOG annotation (%)
MUCL38935	16,174	11123 (68.8)	89.7	7,606 (47.0)	10,968 (67.8)
SHA17-1	12,850	10228 (79.6)	97.4	6,119 (47.6)	9,078 (70.6)
SL200	12,800	10338 (80.8)	96.9	6,203 (48.5)	8,932 (69.8)
SL198	13,453	10553 (78.4)	94.8	6,334 (47.1)	9,233 (68.6)

*FPKM > 1

References:

1. Balasundaram SV, Hess J, Durling MB, Moody SC, Thorbek L, Progida C, et al. The fungus that came in from the cold: dry rot's pre-adapted ability to invade buildings. *ISME J* 2018; **12**: 791–801.

2. Bolger AM, Lohse M, Usadel B. Trimmomatic: a flexible trimmer for Illumina sequence data. *Bioinformatics* 2014; **30**: 2114–2120.

3. Zerbino DR, Birney E. Velvet: Algorithms for de novo short read assembly using de Bruijn graphs. *Genome Res* 2008; **18**: 821–829.

4. Simão FA, Waterhouse RM, Ioannidis P, Kriventseva EV, Zdobnov EM. BUSCO: assessing genome assembly and annotation completeness with single-copy orthologs. *Bioinformatics* 2015; **31**: 3210–3212.

5. Kim D, Paggi JM, Park C, Bennett C, Salzberg SL. Graph-based genome alignment and genotyping with HISAT2 and HISAT-genotype. *Nat Biotechnol* 2019; **37**: 907–915.

6. Grabherr MG, Haas BJ, Yassour M, Levin JZ, Thompson DA, Amit I, et al. Trinity: reconstructing a full-length transcriptome without a genome from RNA-Seq data. *Nat Biotechnol* 2011; **29**: 644–652.

7. Haas BJ, Salzberg SL, Zhu W, Pertea M, Allen JE, Orvis J, et al. Automated eukaryotic gene structure annotation using EVidenceModeler and the Program to Assemble Spliced Alignments. *Genome Biol* 2008; **9**: R7.

8. Stanke M, Diekhans M, Baertsch R, Haussler D. Using native and syntenically mapped cDNA alignments to improve de novo gene finding. *Bioinforma Oxf Engl* 2008; **24**: 637–644.

9. Hoff KJ, Lange S, Lomsadze A, Borodovsky M, Stanke M. BRAKER1: Unsupervised RNA-Seq-Based Genome Annotation with GeneMark-ET and AUGUSTUS. *Bioinforma Oxf Engl* 2016; **32**: 767–769.

10. Testa AC, Hane JK, Ellwood SR, Oliver RP. CodingQuarry: highly accurate hidden Markov model gene prediction in fungal genomes using RNA-seq transcripts. *BMC Genomics* 2015; **16**: 170.

11. Jones P, Binns D, Chang H-Y, Fraser M, Li W, McAnulla C, et al. InterProScan 5: genome-scale protein function classification. *Bioinformatics* 2014; **30**: 1236–1240.

12. Eddy SR. Accelerated Profile HMM Searches. *PLoS Comput Biol* 2011; **7**: e1002195.

13. Finn RD, Bateman A, Clements J, Coggill P, Eberhardt RY, Eddy SR, et al. Pfam: the protein families database. *Nucleic Acids Res* 2014; **42**: D222-230.

14. Huerta-Cepas J, Forslund K, Coelho LP, Szklarczyk D, Jensen LJ, von Mering C, et al. Fast Genome-Wide Functional Annotation through Orthology Assignment by eggNOG-Mapper. *Mol Biol Evol* 2017; **34**: 2115–2122.

15. Huerta-Cepas J, Szklarczyk D, Forslund K, Cook H, Heller D, Walter MC, et al. eggNOG 4.5: a hierarchical orthology framework with improved functional annotations for eukaryotic, prokaryotic and viral sequences. *Nucleic Acids Res* 2016; **44**: D286–D293.

16. Lombard V, Golaconda Ramulu H, Drula E, Coutinho PM, Henrissat B. The carbohydrate-active enzymes database (CAZy) in 2013. *Nucleic Acids Res* 2014; **42**: D490-495.

17. Dobin A, Davis CA, Schlesinger F, Drenkow J, Zaleski C, Jha S, et al. STAR: ultrafast universal RNA-seq aligner. *Bioinforma Oxf Engl* 2013; **29**: 15–21.

18. Hartley SW, Mullikin JC. QoRTs: a comprehensive toolset for quality control and data processing of RNA-Seq experiments. *BMC Bioinformatics* 2015; **16**: 224.

19. Love MI, Huber W, Anders S. Moderated estimation of fold change and dispersion for RNA-seq data with DESeq2. *Genome Biol* 2014; **15**: 550.

20. Löytynoja A. Phylogeny-aware alignment with PRANK. *Methods Mol Biol Clifton NJ* 2014; **1079**: 155–170.

21. Chang J-M, Di Tommaso P, Notredame C. TCS: a new multiple sequence alignment reliability measure to estimate alignment accuracy and improve phylogenetic tree reconstruction. *Mol Biol Evol* 2014; **31**: 1625–1637.

22. Darriba D, Taboada GL, Doallo R, Posada D. ProtTest 3: fast selection of best-fit models of protein evolution. *Bioinforma Oxf Engl* 2011; **27**: 1164–1165.

23. Stamatakis A. RAxML version 8: a tool for phylogenetic analysis and post-analysis of large phylogenies. *Bioinformatics* 2014; **30**: 1312–1313.

24. Wu Y-C, Rasmussen MD, Bansal MS, Kellis M. TreeFix: Statistically Informed Gene Tree Error Correction Using Species Trees. *Syst Biol* 2013; **62**: 110–120.

25. Wu Y-C, Rasmussen MD, Bansal MS, Kellis M. Most parsimonious reconciliation in the presence of gene duplication, loss, and deep coalescence using labeled coalescent trees. *Genome Res* 2014; **24**: 475–486.

26. Huerta-Cepas J, Serra F, Bork P. ETE 3: Reconstruction, Analysis, and Visualization of Phylogenomic Data. *Mol Biol Evol* 2016; **33**: 1635–1638.
